# Comparison of the 6th and 7th Editions of the UICC-AJCC TNM Classification for Esophageal Cancer

**DOI:** 10.1245/s10434-012-2218-5

**Published:** 2012-03-07

**Authors:** Koen Talsma, Pieter van Hagen, Brechtje A. Grotenhuis, Ewout W. Steyerberg, Hugo W. Tilanus, Jan J. B. van Lanschot, Bas P. L. Wijnhoven

**Affiliations:** 1 Department of Surgery, Erasmus Medical Center, Rotterdam, The Netherlands; 2 Department of Public Health, Erasmus Medical Center, Rotterdam, The Netherlands

## Abstract

**Background:**

The new 7th edition of the Union for International Cancer Control–American Joint Committee on Cancer (UICC-AJCC) tumor, node, metastasis (TNM) staging system is the ratification of data-driven recommendations from the Worldwide Esophageal Cancer Collaboration database. Generalizability remains questionable for single institutions. The present study serves as a validation of the 7th edition of the TNM system in a prospective cohort of patients with predominantly adenocarcinomas from a single institution.

**Methods:**

Included were patients who underwent transhiatal esophagectomy with curative intent between 1991 and 2008 for invasive carcinoma of the esophagus or gastroesophageal junction. Excluded were patients who had received neoadjuvant chemo(radio)therapy, patients after a noncurative resection and patients who died in the hospital. Tumors were staged according to both the 6th and the 7th editions of the UICC-AJCC staging systems. Survival was calculated by the Kaplan–Meier method, and multivariate analysis was performed with a Cox regression model. The likelihood ratio chi-square test related to the Cox regression model and the Akaike information criterion were used for measuring goodness of fit.

**Results:**

A study population of 358 patients was identified. All patients underwent transhiatal esophagectomy for adenocarcinoma. Overall 5-year survival rate was 38%. Univariate analysis revealed that pT stage, pN stage, and pM stage significantly predicted overall survival. Prediction was best for the 7th edition, stratifying for all substages.

**Conclusions:**

The application of the 7th UICC-AJCC staging system results in a better prognostic stratification of overall survival compared to the 6th edition. The fact that the 7th edition performs better predominantly in patients with adenocarcinomas who underwent a transhiatal surgical approach, in addition to findings from earlier research in other cohorts, supports its generalizability for different esophageal cancer practices.

Accurate staging of cancer is important for stage-specific treatment, thus minimizing inappropriate treatment. Moreover, it allows for interinstitutional comparisons and disclosure of prognosis to patients.^1^ The staging system for cancer in the esophagus and esophagogastric junction has been revised as outlined in the 7th edition of the Union for International Cancer Control/Union Internationale Contre le Cancer (UICC) and the American Joint Committee on Cancer (AJCC), Cancer Staging Manual.^2^

Retrospective studies suggested that the number of involved lymph nodes is a better predictor of outcome than classifying lymph node involvement as either present or absent.^3^,^4^ Peyre et al. showed that patients with ≥3 lymph nodes involved have a risk of systemic disease that exceeds 50%. When >8 nodes are involved, the risk of dying is almost 100%.^5^ Indeed, the latest 7th edition of the UICC-AJCC esophageal tumor, node, metastasis (TNM) staging system has acknowledged the importance of the number of involved nodes by revising the N category from site-dependent staging to a numerically based classification into N0 to N3. Another major change is the definition of regional lymph nodes.

The new UICC-AJCC staging system is the ratification of data-driven recommendations from a database of >7800 esophageal cancer patients created from a large multi-institutional collaboration involving 13 institutions.^6^,^7^ This Worldwide Esophageal Cancer Collaboration (WECC) database overcomes problems of rarity of this cancer, but generalizability remains questionable for single institutions. WECC incorporates high-volume centers both from the West (where adenocarcinomas prevail) and from the East (where most tumors are squamous cell carcinomas). Moreover, the extent of intrathoracic lymph node dissection can vary greatly between different institutions, leading to potential bias.

The present study serves as a validation of the WECC-based 7th edition of the TNM system in a cohort of patients with both squamous cell carcinomas and adenocarcinomas from a single Western high-volume institution. Two studies already showed that the 7th edition criteria resulted in better prognostic stratification than the 6th edition.^8^,^9^ However, both study cohorts consisted of squamous cell carcinomas or junctional tumors, respectively. Moreover, Gaur et al. included patients who received (neo)adjuvant therapy.^9^

The aim of this study was to assess the predictive ability of the 7th edition of the AJCC TNM staging system for overall survival and to compare this with the 6th edition in a cohort of patients who underwent transhiatal esophagectomy for adenocarcinomas without (neo)adjuvant therapy.

## Patients and Methods

### Study Population

Included were all patients who underwent a transhiatal esophagectomy with curative intent between January 1991 and September 2008 at the Erasmus Medical Center (Rotterdam, The Netherlands) for invasive squamous cell carcinoma and adenocarcinoma of the esophagus or gastroesophageal junction. Excluded were patients who had received neoadjuvant chemo(radio)therapy, patients after a noncurative (R1) resection (tumor-free margin <1 mm) and patients who died in the hospital. Clinicopathologic data of all patients had been routinely collected in an ongoing prospective registry.

### Surgery

Transhiatal esophagectomy with cervical anastomosis was the chosen surgical approach in the present study. This encompasses the en-bloc dissection of the primary tumor and its adjacent lymph nodes under direct vision through the widened hiatus of the diaphragm up to the level of the inferior pulmonary vein. Subsequently, a 3–4-cm-wide gastric tube is created. The left gastric artery is transected at its origin with resection of celiac trunk lymph nodes. After mobilization and transection of the cervical esophagus, the intrathoracic middle and upper esophagus is bluntly dissected in an antegrade fashion with a vein stripper. Esophagogastrostomy is performed in the neck without a formal cervical lymphadenectomy.

### Follow-up

Surviving patients were followed at regular intervals at the outpatient clinic until 5 years after surgery. Outpatient clinic visits encompassed history taking and physical examination. No routine imaging was performed. Recurrences were sought afterward, only when clinically indicated, by CT scan or ultrasound and proven by histology and cytology whenever possible. Overall survival was defined as the time between date of operation and date of death. Surviving patients were censored on the day of last follow-up. Patient survival status was calculated after contacting the general practitioners (performed by a trained data manager). The last follow-up checkpoint was July 2010. If follow-up was incomplete, survival was verified in the municipal mortality registers.

### Statistical Analysis

Tumors were staged according to both the 6th and 7th editions of the UICC-AJCC staging systems. Survival was calculated by the Kaplan–Meier method, and differences between curves were assessed by the log rank test.

Two multivariable models were built, one with the 6th edition and one with the 7th edition of the TNM staging system as categorical variables. The performance was tested for the model in which the stages were combined into four categories (I–IV) as well as for the model with all substages included (IA, IB, IIA, IIB, IIIA, IIIB, IIIC, IV). A multivariable model with both 6th and 7th edition criteria included was used to assess the remaining value of the 6th edition when the 7th edition information was known.

The likelihood ratio chi-square test related to the Cox regression model was used for measuring goodness of fit. The Akaike information criterion (AIC) was applied to correct for the potential bias in comparing prognostic systems with different number of stages.^10^,^11^ The −2 log likelihood (which is the parameter in the Cox regression) of the 6th edition was compared to that of the 7th edition; the smaller the value of this statistic, the better the model.

AIC was defined as: AIC = −2 log maximum likelihood + 2 × (the number of parameters in the model). A smaller AIC value indicates a more desirable model for predicting outcome. A value of *P* < 0.05 was considered statistically significant. Statistical analysis was performed with SPSS 10 for Windows (SPSS, Chicago, IL).

## Results

### Patient Characteristics

A consecutive series of 766 patients underwent esophagectomy with curative intent. In total, 221 patients were excluded because they had received neoadjuvant chemo(radio)therapy in the context of a randomized, controlled trial.^12^ Another 165 patients were excluded because of a noncurative (R1) resection, and 20 patients were excluded because of in-hospital mortality. Two patients had an in situ carcinoma and were also excluded from the current analysis. This resulted in a final study population of 358 patients.

Mean follow-up was 51 months (median 37 months). Overall 5-year survival rate was 38%. Most recurrences of disease occurred within 2 years after surgery.

Patient characteristics and overall survival rates are summarized in Table 1. All patients underwent transhiatal esophagectomy for adenocarcinoma. Eight patients seemed to have distant metastasis during the operation; their disease was scored as M1.

**Table 1:** Patient demographics and results of univariate analysis for overall survival (*N* = 358)

Characteristic	Value	5-y survival, %	*P*
No. of patients	358		
Age, year, mean (range)	62.6 (28–83)	38.8	
Gender			
Male	293 (82%)	37.2	0.664
Female	65 (18%)	45.9	
pT			
1	78 (22%)	68.7	<0.001
2	79 (22%)	51.1	<0.001
3	201 (56%)	22.7	
pN			
0	146 (41%)	65.9	<0.001
1	90 (25%)	28.4	<0.001
2	81 (23%)	17.5	<0.001
3	41 (11%)	3.0	
pM			
0	350	39.7	<0.001
1	8	0.0	
Grade			
Well differentiated (G1)	31 (9%)	75.3	<0.001
Moderately differentiated (G2)	177 (49%)	39.4	<0.053
Poorly differentiated (G3)	150 (42%)	30.9	
Histology			
Squamous cell carcinoma	47 (13%)	41.9	0.752
Adenocarcinoma	311 (87%)	38.3	
Location			
Upper third	6 (2%)	30.4	0.352
Middle third	14 (4%)	42.6	0.325
Lower third (distal + EGJ)	338 (94%)	36.9	
Type of surgical approach			
Transhiatal esophagectomy	358 (100%)		
Transthoracic esophagectomy			

*T* tumor stage (depth of invasion), *N* lymphatic dissemination stage (according to 7th edition of UICC-AJCC TNM staging system: *N0* no positive lymph nodes, *N1* 1–2 positive lymph nodes, *N2* 3–6 positive lymph nodes, *N3* ≥6 positive lymph nodes), *M* distant metastasis stage (according to 7th edition of UICC-AJCC TNM staging system: *M0* no metastasis, *M1* distant metastasis present), *EGJ* esophagogastric junction

Univariate analysis revealed that parameters pT stage, pN stage, and pM stage all significantly predicted overall survival. Except for histologic grade, no other significant predictors of survival were detected in this univariate analysis. The median number of dissected nodes per patient was 11. In patients with negative lymph nodes (pN0), the survival rates did not differ between patients with ≤11 nodes and >11 nodes dissected: 65% vs. 69%, respectively; *P* = 0.65; data not shown).

### Stratification of Prognosis According to 6th and 7th Editions of TNM Staging Systems

**Fig. 1:**
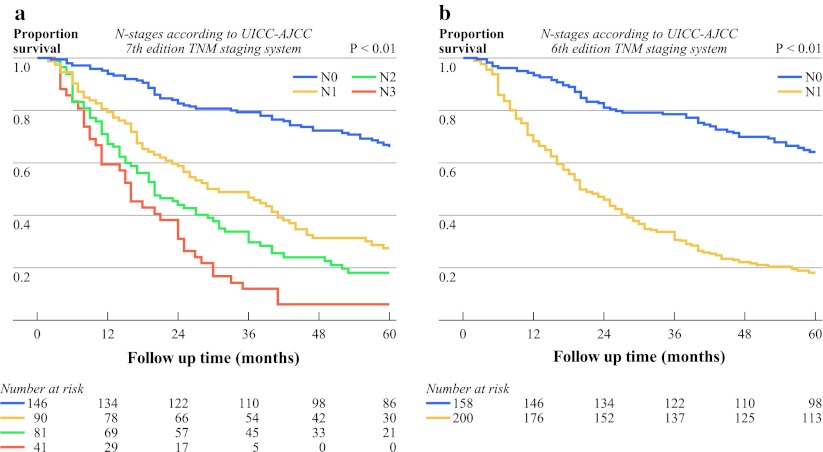
Kaplan–Meier overall survival curves for 358 patients stratified by N stage according to **a** 7th edition and **b** 6th edition UICC-AJCC TNM staging systems (overall log rank *P* < 0.01)

The overall survival curves according to the N classifications of the 6th and 7th editions are shown in Fig. 1a and b, respectively.

Patient stage migration for reclassifying patients from the 6th to the 7th staging system and their survival rates are listed in Table 2. In 58% of the 358 esophageal cancer patients, stage did not differ in these two classification systems. Reassignment of disease stage occurred in all other patients, either to a higher or to a lower tier. According to the 6th edition staging system, 56 (87%) of 64 stage IV patients were staged as such because of a celiac lymph node metastasis. These patients were reclassified to a lower tier in the 7th edition: 6 of 64 were staged as stage IIB, 15 as stage IIIA, 19 as IIIB, and 16 as IIIC (Table 2).

**Table 2:** Cross table of staging esophageal cancer patients according to the 6th and 7th editions of UICC-AJCC TNM staging

6th edition^a^						5 year-survival according to 7th edition (%)
	I	IIA	IIB	III	IV	
7th edition^b^						
IA	43	0	0	0	0	87.7
IB	13	28	0	0	0	73.3
IIA	0	19	0	0	0	55.3
IIB	0	41	24	0	6	40.1
IIIA	0	0	21	50	15	24.3
IIIB	0	0	0	31	19	11.9
IIIC	0	0	4	20	16	3.1
IV	0	0	0	0	8	0.0
5 year-survival according to 6th edition (%)	81.9	56.8	38.3	14.1	12.4	

*M1a* celiac nodes involved in lower esophageal cancer or cervical nodes involved in upper esophageal cancer, *M1b* beyond locoregional node involvement (i.e., cervical nodes in lower esophageal cancer and celiac nodes in upper esophageal cancer; metastatic involvement of visceral organs, pleura, peritoneum)

^a^The 6th edition AJCC-UICC TNM staging system: *stage I* T_1_N_0_, *stage IIA* T_2,3_N_0_, *stage IIB* T_1,2_N_1_, *stage III* T_3_N_1_ or T_4_N_0_, *stage IVA* T_any_N_any_M1a, *stage IVB* T_any_N_any_M1b. The 7th edition AJCC-UICC TNM staging system (for adenocarcinoma): *stage IA* T_1_N_0_G_1,2_, *stage IB* T_1_N_0_G_3_ or T_2_N_0_G_1,2_, *stage IIA* T_2_N_0_, *stage IIB* T_3_N_0_ or T_1,2_N_1_, *stage IIIA* T_4_N_0_ or T_3_N_1_ or T_1,2_N_2_, *stage IIIB* T_3_N_2_, *stage IIIC* T_any_N_3_ or T_4a_N_1–3_ or T_4b_N_any_, *stage IV* T_any_,N_any_,M_1_

**Fig. 2:**
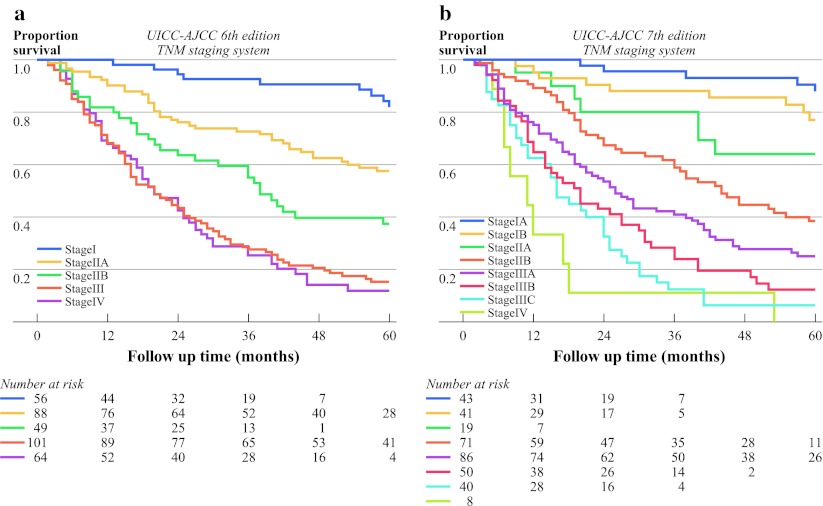
Kaplan–Meier curves of overall survival for 358 patients stratified according to **a** 6th edition and **b** 7th edition UICC-AJCC TNM staging systems

The Kaplan–Meier curves of esophageal cancer patients based on the 6th and 7th editions of the TNM staging systems are depicted in Fig. 2. Both systems show a relatively ordered monotone distribution of survival. However, according to the 6th edition staging system, the Kaplan–Meier plot shows overlapping curves for stage III and IV. In the 7th edition, no important overlapping occurs among stages I through IV.

**Fig. 3:**
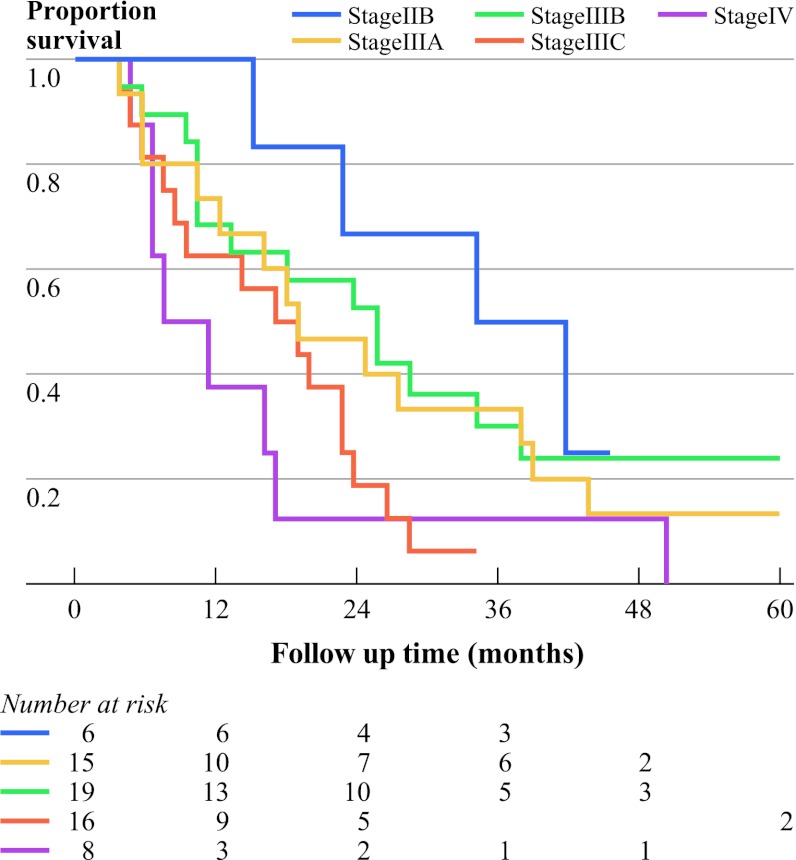
Kaplan–Meier overall survival curves for 64 UICC-AJCC 6th stage IV patients who were reclassified according to UICC-AJCC 7th edition TNM staging (log rank *P* = 0.43)

Subgroup analysis among selected patients who had been considered to have stage IV disease acording to the UICC-AJCC 6th edition scoring system showed that patients reclassified from stage IV disease to a lower tier in the UICC-AJCC 7th edition had a significantly better survival compared to patients still classified as stage IV according to the UICC-AJCC 7th edition. Moreover, the UICC-AJCC 7th edition was able to make further significant stratification of survival rates of these reclassified patients (Fig. 3; log rank *P* = 0.43).

The UICC-AJCC 7th edition staging system defines patients with positive paraesophageal cervical lymph nodes (*n* = 10) as having stage IIIA or IIIB disease. These patients, however, had a prognosis as bad as that of patients with distant metastasis (1-year overall survival rate 30% vs. 33%).

The performance of the 6th and 7th edition staging systems won quantified by the likelihood ratio chi-square and AIC (Table 3). Predictive ability was best for the full 7th edition criteria stratifying for all substages (highest likelihood ratio χ^2^). AIC value was smaller for the 7th edition compared to the 6th edition staging system, indicating that it has a better prognostic stratification. The AIC value was lowest when patients with cervical lymph node metastasis at a large distance from the primary tumor (i.e., the lower third of the esophagus) were also classified as having stage IV disease. When the 6th and 7th edition staging systems are both included in one Cox regression model, the 6th edition no longer significantly predicted survival, whereas the 7th edition remained a significant stratifier of prognosis (data not shown).

**Table 3:** Prognostic stratification of the 6th and 7th editions of the UICC-AJCC TNM staging systems

Model	Figure	Subgroups	LR χ^2^	AIC value^a^
6th edition	2a	I, II, III, IV	96.9	2607.1
7th edition, full	2b	IA, IB, IIA, IIB, IIIA, IIIB, IIIC, IV	128.6	2592.9
7th edition, collapsed		I, II, III, IV	99.0	2605.4

*AIC* Akaike information criteria, *LR* likelihood ratio

^a^A lower AIC value represents a better discriminatory model

## Discussion

This study shows that both the 6th and 7th UICC-AJCC TNM staging systems have a distinctive and monotone (ordered) relationship of stage group to overall survival for esophageal cancer patients who have undergone potentially curative surgery without (neo)adjuvant therapy. Distribution of patients among different stages is in line with that described in the literature. All groups are large enough for proper statistical analysis, except for stage IIA in the 7th edition.

Further testing of both systems on the present data shows that the 7th edition has the best performance because of the lowest AIC (i.e., a better fit) when Cox regression models are used. Survival curves stratified according to the UICC-AJCC 7th edition TNM staging system did not overlap, which is in contrast to the curves of the 6th edition. Moreover, further stratification of N stage according to number of positive lymph nodes in the 7th edition is indeed valuable, as shown in Fig. 1.

A major change in the new TNM staging system is the definition of regional lymph nodes. There has always been debate regarding the prognostic importance of positive celiac nodes, which were considered distant metastases in earlier editions.^13^ In the 6th edition staging system, the Kaplan–Meier plot showed overlapping curves for stage III and IV. According to the UICC-AJCC 7th edition, only patients with distant metastasis can be categorized as having stage IV disease. In contrast, according to the 6th edition, most stage IV disease was due to nonregional celiac lymph node metastasis, whereas stage IIB and III consisted of regional lymph node metastasis. Hence, 87% (56 of 64) of the patients with stage IV disease who were assessed according to the 6th edition criteria were reclassified as having stage IIB, IIIA, IIIB, and IIIC disease according to 7th edition criteria. Because these stages all had different survivals (Fig. 3), the present results support the new concept that it is unnecessary to identify nonregional lymph node metastasis and to label these nodes as M1A or M1B.

Two previous studies have compared the performance of 6th with the 7th editions of the TNM staging system in predicting survival. Hsu et al. evaluated 392 patients who underwent primary surgical resection through a tri-incisional approach in Taiwan during 1995–2006.^8^ In the other study, nearly two-thirds of the patients received neoadjuvant therapy.^9^

Both Hsu et al. and Gaur et al. concluded that the 7th edition of the staging system was a better model for predicting outcome.^8^,^9^ The most important difference with the present study is tumor histology; the vast majority of our patients had an adenocarcinoma, and almost all patients underwent a transhiatal resection.

The WECC-based 7th edition of the TNM staging system was built on data from patients without neoadjuvant treatment in a squamous cell carcinoma predominant database. Our sample population from a single institution is of course small compared with the worldwide esophageal cancer collaboration database, but the surgical procedures were highly uniform throughout the entire study period. The previous studies of Hsu et al. and Gaur et al., as well as the present study, underline the generalizability of the 7th edition and make it broadly applicable for daily clinical practice of esophageal cancer surgery around the world.^8^,^9^

The 7th edition of the UICC-AJCC esophageal TNM staging system has acknowledged the importance of the number of involved nodes by subdividing the N classification into N0 to N3. The transhiatal approach may profoundly affect the completeness of lymph node dissection and, accordingly, proper nodal staging. On the basis of data from a Dutch trial, nowadays, tumors proximal of esophagogastric junction (Siewert type 1) are preferably offered a transthoracic approach in our institution.^14^,^15^ The latter approach will result in the collection of more lymph nodes and might give a more valid node sampling for staging. To which extent lymph nodes should be sampled for proper staging remains an important issue.^16^ In a study performed by Peyre et al., the number of lymph nodes removed was an independent predictor of survival and a minimum number of 23 regional lymph nodes was proposed.^17^ In the present study, the median number of nodes removed in a transhiatal approach was 11. This relatively scarce lymph node collection result can be seen as a drawback of our study, but it also gives rise to a remarkable finding. Although all patients underwent a transhiatal esophagectomy, the survival curves of different N stages (N0–N3; Fig. 1) do not overlap in our data, which probably indicates that there has been a valid and robust node sampling. On the other hand, there seems to be a relatively large difference in survival rate between N0 and N1. We know from previous studies that there is a dichotomy in survival rate between tumors that did and did not lymphatically disseminate.^18^ Early tumors (pT1) with lymph node invasion have prognosis comparable to tumors with more advanced T stage. Lymphatic dissemination is an independent indication of the biological aggressiveness of the tumor.

However, the large step in survival rate between N0 and N1 might also be due to a stage migrational effect. This, the so-called Will Rogers effect, means that stage N1 disease might actually include N2 or even N3 disease as a result of invalid node sampling.^19^ The WECC group has indicated a resection of a minimum of 10 nodes for T1, 20 for T2, and ≥30 nodes for T3–4 to be resected to obtain optimal results.^20^ In N0 patients, such an effect does not occur; we found no significant difference in survival rates according to the number of resected lymph nodes in lymph node–negative patients. However, a median of 11 nodes definitely entails the risk of a stage migration effect in the patient group with positive nodes.

Finally, an important question remains: does a better predictive staging system have consequences for preoperative decision making? Medical decision making in terms of administering neoadjuvant chemotherapy and choosing the optimal surgical approach for esophagectomy is often based on clinical N staging. Lack of accurate preoperative staging is a major problem in allocating treatment modalities in these patients. It has been recently shown that further stratification according to the position of the positive node relative to the diaphragm can effectively discriminate between node-positive patients.^21^ The overall accuracy for endoscopic ultrasound and CT in predicting the N stage per station is moderate, however. When the therapeutic approach depends on the status of a specific lymph node station, a more objective and reliable assessment of lymph nodal involvement (e.g., endoscopic ultrasound–fine-needle aspiration) should be considered.^22^

This study indicates that the application of the 7th UICC-AJCC staging system results in a better prognostic stratification of overall survival compared to the 6th edition. The fact that the 7th edition also has a superior prognostic ability in this study population from a single high-volume institution with predominantly adenocarcinomas and a two-incisional surgical approach supports its generalizability for different esophageal cancer practices.
